# A Coupled EBSD/TEM Analysis of the Microstructure Evolution of a Gradient Nanostructured Ferritic/Martensitic Steel Subjected to Surface Mechanical Attrition Treatment

**DOI:** 10.3390/ma12010140

**Published:** 2019-01-03

**Authors:** Wenbo Liu, Xiao Jin, Bo Zhang, Di Yun, Piheng Chen

**Affiliations:** 1 Department of Nuclear Science and Technology, Xi’an Jiaotong University, Xi’an 710049, China; liuwenbo@xjtu.edu.cn (W.L.); bzhang_nss@xjtu.edu.cn (B.Z.); 2 Suzhou Nuclear Power Research Institute, Suzhou, Jiangsu 215004, China; jin_xiao2002@163.com; 3 Science and Technology on Surface Physics and Chemistry Laboratory, P.O. Box 9071-35, Jiangyou 621907, China

**Keywords:** severe plastic deformation, reduced ferritic/martensitic steel, misorientation distribution, EBSD

## Abstract

Surface mechanical attrition treatment (SMAT) was performed on a reduced ferritic/martensitic (RAFM) steel to form a nanostructured (NS) layer on the surface of the sample. Both electron backscatter diffraction (EBSD) and TEM were used to investigate the microstructure evolution during SMAT. The experimental results showed that there were three different zones after SMAT: (i) The “ultrafine grain” (UFG) zone, observed at the top-most surface region, (ii) the “transition zone” in which the original grains were fragmented under the severe plastic deformation and (iii) the “deformed zone” in which the original grains were simply deformed. The average grain sizes increased rapidly with the increase of depth, while the Vickers hardness decreased with the increase of depth, and this phenomenon could be explained in terms of boundary strengthening and dislocation strengthening. The number fractions of medium-angle grain boundaries (MAGBs) and medium-high-angle grain boundaries (MHAGBs) in UFG zones were larger than those in the transition zone and the deformed zone. However, the number fraction of the low-angle grain boundaries (LAGBs) was extremely small in all the zones after SMAT, especially in the transition zone.

## 1. Introduction

In the past several decades, much attention has been paid to the development and application of processes for producing gradient nanostructured (NS) materials on the surface layers of alloys and metals, using severe plastic deformation (SPD) [1,2], such as: High-pressure torsion (HPT) [3], simple shear extrusion (SEE) [4], equal channel angular pressing (ECAP) [5], accumulative rolling bonding (ARB) [6], and surface mechanical attrition treatment (SMAT) [7]. Among these methods, SMAT (sometimes entitled ultrasonic shot peening (USSP)) has been proved as a novel and efficient technique to produce bulk NS materials, and grain refinement mechanism during SMAT has been systematically studied using transmission electron microscopy (TEM) [8,9], in which phase transformations and dislocation activities were found to make key roles. However, the observation of microstructure evolution using TEM was still restricted due to the limited observation area. Hence, a comprehensive understanding of the microstructure evolution and deformation mechanisms during SMAT is still of paramount importance for understanding the development and application of NS materials.

Electron backscatter diffraction (EBSD) is regarded as an effective tool to investigate the microstructure evolution without area restriction and can be used to provide detailed misorientation information on the deformed structural. By mainly using EBSD, several studies have been performed to characterize textures, grain sizes and morphology, crystallographic orientation, and grain boundary (GB) misorientation and character after SPD [10,11,12,13]. However, only a few works have focused on the microstructure characterization after SMAT [9,13,14], since the depth of the deformed layer and the range of the surface gradient NS materials appears limited [15]. Moreover, a quantitative description of the microstructure evolution during SMAT, would enable depiction—both rapidly and accurately—of the effect of the processing parameters [14]. Compared to TEM, however, the EBSD technology has a relatively lower special resolution. Thus, a combined EBSD/TEM analysis can provide a significant advantage to understand the microstructure evolution during SMAT. 

In the present work, a gradient NS layer on reduced ferritic/martensitic (RAFM) steel was produced by SMAT. EBSD and TEM were then used to investigate the microstructure evolution during SMAT, and the Vickers microhardness test was used for understanding the relationship between microstructure and strength during SMAT. The GB misorientations in different zones after SMAT were also systematically analyzed, based on the EBSD results.

## 2. Experiments

The steel used in the present work was a reduced ferritic/martensitic (RAFM) steel with a chemical composition (in wt. %): 0.10% C, 8.60% Cr, 0.55% Mn, 1.50% W, 0.09% Ta, 0.29% V and balance Fe. The samples were austenitized at 1253 K for 45 min and then quenched with water. The samples were then tempered at 1033 K for 90 min, before air cooling. Surface mechanical attrition treatment (SMAT) was used to produce a NS layer on the surface of the steel. With the setup illustrated in Lu’s work [16], plate samples (Φ50.0 × 4.0 mm in size) of the tempered steel were subjected to SMAT. With a vibration frequency of 20 kHz, the plate samples were treated for 30 min and the ball size was 5 mm in diameter, which was similar to our previous work [17].

In order to investigate the gradient microstructure after SMAT, the sample was examined in the cross-section using EBSD mapping in a scanning electron microscope (SEM, TESCAN MIRA3, Czech) equipped with a field-emission gun. The sample was first subjected to grinding and mechanical polishing. A step size of 100 nm was chosen during the EBSD test to investigate the finest scale structures in the topmost surface of the sample after SMAT. Cross-sectional observation of the samples after heat treatment was performed on TEM of type JEOL Tecnai F20 (Akishima, Tokyo). The means of a focused ion beam (FIB) were used to produce the cross-sectional TEM samples. The mechanical strength in the surface layer after SMAT was evaluated using a Vickers microhardness indenter of type MH-5L, taking measurements by applying 10 g for 5 s of dwell time on a polished cross-sectional surface. The hardness tests were repeated 5 times for each point.

## 3. Results and Discussion

### 3.1. Characterization of the Microstructural Morphologies

**Figure 1:**
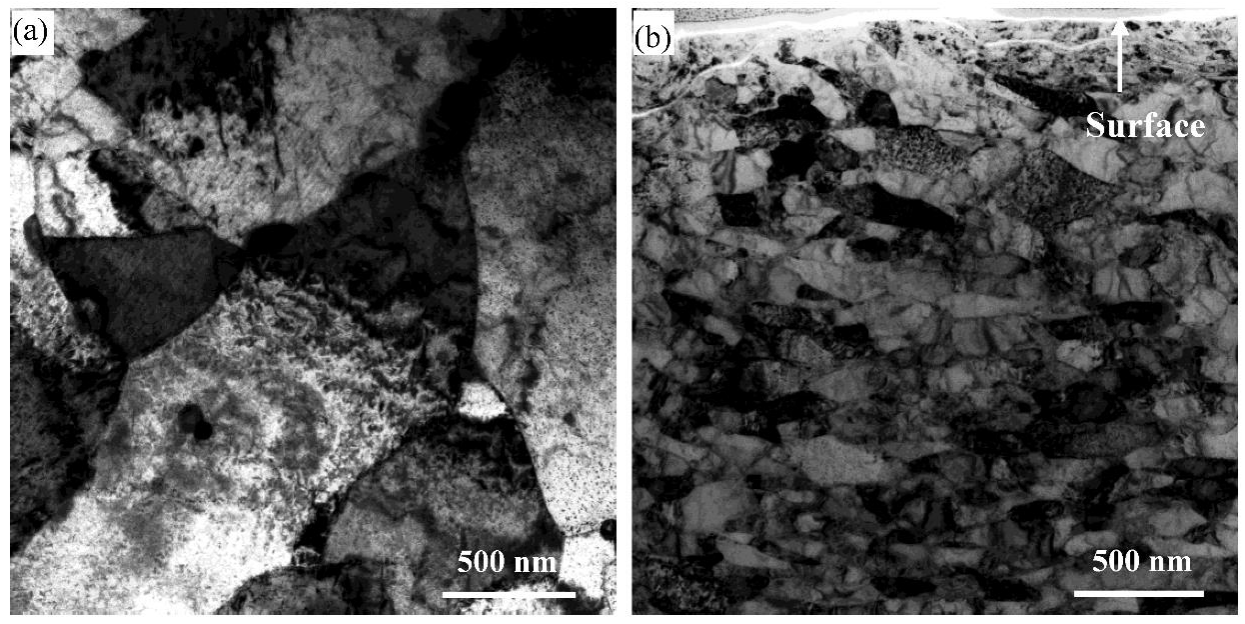
Cross-sectional bright field TEM image of the reduced activation steel (**a**) before and (**b**) after surface mechanical attrition treatment (SMAT).

Cross-sectional bright field TEM images of the reduced activation steel before and after SMAT are shown in Figure 1. Before SMAT, GBs and fine precipitates can be clearly seen (Figure 1a), and the grains with sizes of several micrometers can be observed. The coarse grains had been broken down to NS grains after SMAT (Figure 1b). It was confirmed that the grain size increased gradually with the increase of depth, and extremely fine NS grains were observed in the surface layer. Through statistical calculations, the average grain size after SMAT was 12.6 nm, within the topmost surface.

**Figure 2:**
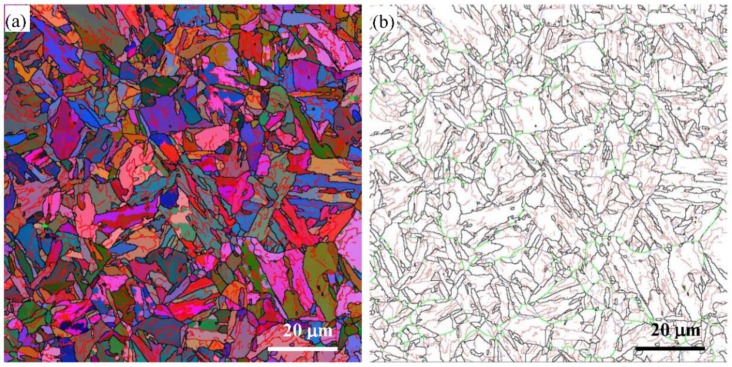
Typical electron backscatter diffraction (EBSD) images of the reduced activation steel before SMAT: (**a**) EBSD orientation color map and (**b**) corresponding boundary misorientation map. The fine red lines mark boundaries with misorientation between 2° and 15°, and the fine blue lines mark boundaries with misorientation between 15° and 30°; while the thick green lines mark boundaries with misorientation between 30° and 45°, and the thick black lines mark boundaries with misorientation larger than 45°.

Typical EBSD images of the reduced activation steel before SMAT, including an inverse pole figure color map and the corresponding boundary map, are shown in Figure 2. The different colors in Figure 2a correspond to the different crystallographic orientations normal to the observed plane [18]. Boundaries with angles lower than 15° are usually classified as low-angle grain boundaries (LAGBs), while boundaries with angles between 15° and 62.8° are usually denoted as high-angle grain boundaries (HAGBs) [11]. As suggested by Bowen [19], further subdivision of the HAGBs were also taken by subdividing them into medium-angle grain boundaries (between 15° and 30°, or MAGBs), medium-high-angle grain boundaries (between 30° and 45°, or MHAGBs) and very-high-angle grain boundaries (between 45° and 62.8°, or VHAGBs). The different colors in Figure 2b corresponded to different misorientation. The fine red lines (LAGBs) were drawn when the misorientation between adjacent points was between 2° and 15°, and the fine blue lines (MAGBs) were drawn when the misorientation between adjacent points was between 15° and 30°; while the thick green lines (MHAGBs) were drawn when the misorientation between adjacent points was between 30° and 45°, and the thick black lines were drawn when the misorientation between adjacent points was larger than 45° (VHAGBs). 

A typical annealed martensitic microstructure with the grain size of several micrometers can be seen in Figure 2a. It is clearly observed from Figure 2b that most of the boundaries before SMAT had misorientations larger than 30° (green lines and black lines). A larger number of boundaries with misorientations smaller than 15° distributed within the annealed tempered martensitic grains (red lines), while the number of boundaries with misorientation between 15° and 30° were very small (blue lines). Most of the grain boundaries are HAGBs. With regard to the grain boundaries and the grain size, the microstructure before SMAT is homogenous.

**Figure 3:**
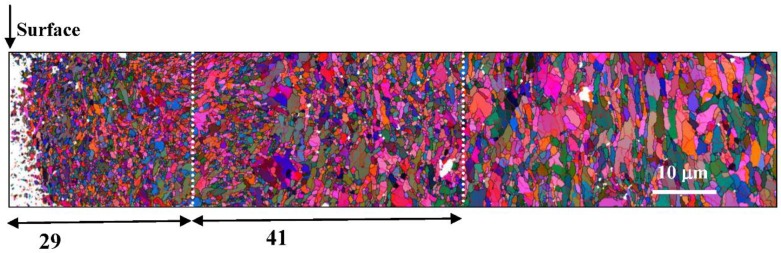
Cross-sectional EBSD orientation color map of the reduced activation steel after SMAT.

A cross-sectional EBSD orientation color map of the reduced activation steel after SMAT is shown in Figure 3. Here, the grain sizes changed gradually with the increase of depth after SMAT. In the topmost surface, within a depth of about 29 μm, extremely fine equiaxed grains were observed; while nanoscale lamellas, parallel to the treated surface, were observed where the depth was larger than about 70 μm. However, the grains in the region with depth between 29 μm and 70 μm were also very small, although a few equiaxed grains with slightly larger sizes were observed in this region.

### 3.2. Correlation between Vickers Hardness and Microstructure Features

**Figure 4:**
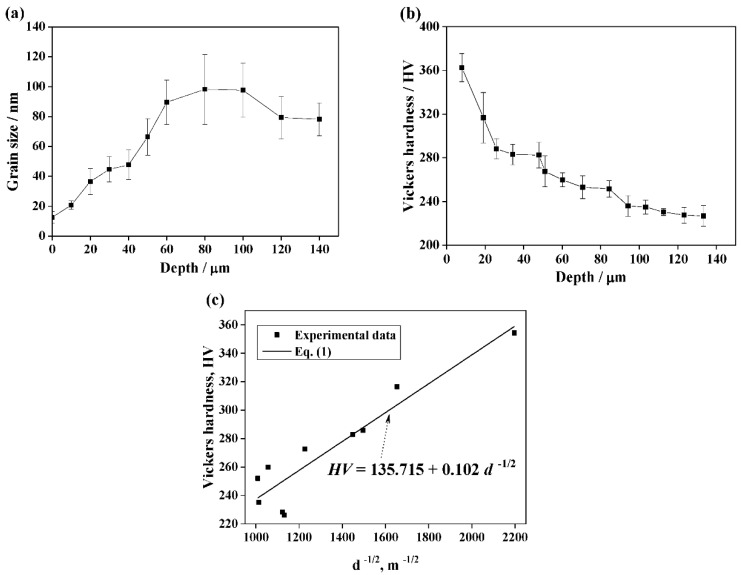
Average grain size evolution (**a**) and Vickers hardness evolution (**b**) in function of depth from the surface, (**c**) variation of Vickers hardness with *d*^−1/2^ using experimental data and Equation (1).

The average grain size evolution obtained from the TEM images and the Vickers hardness evolution in function of depth from the treated surface are shown in Figure 4. The average grain size increased rapidly with the increase of depth when the depth was smaller than 80 μm, and a slight decrease of average grain size was observed when the depth was larger than 100 μm. However, the Vickers hardness in the topmost surface of the treated sample was the highest and decreased with the increase of depth. It was reported that hardness linearly correlates with tensile strength [20], and that hardness is affected by the microstructure, in addition to grain size [21]. 

**Figure 5:**
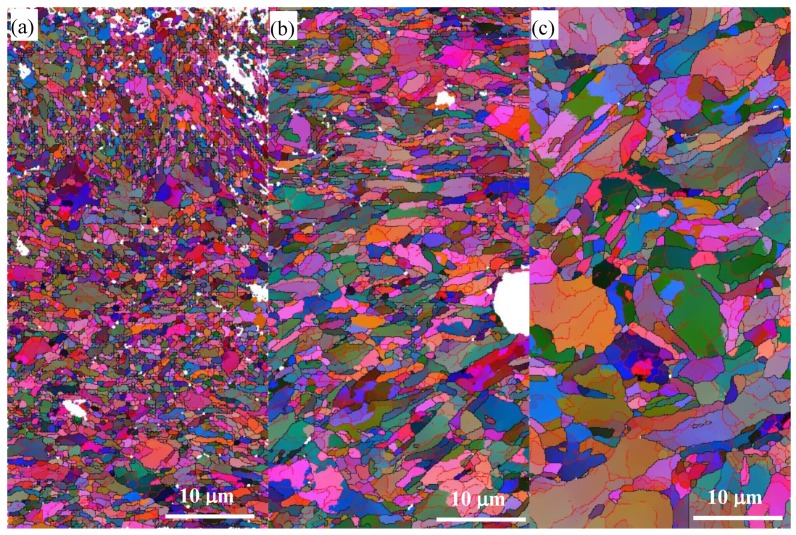
Typical EBSD orientation color maps of the reduced activation steel after SMAT: (**a**) The ultrafine grain zone, (**b**) the transition zone and (**c**) the deformed zone.

The strength-structure relationship can usually be described in terms of boundary strengthening and dislocation strengthening for the structures consisting of interconnecting boundaries and lamellar boundaries [21]. The Hall–Petch strengthening method can be used to calculate the strength contribution of the boundaries with misorientations larger than 15° [21,22]. The strength contribution of the boundaries with a misorientation angle smaller than 15° may depend on the boundary misoriention angle, since the strength contribution is proportional to the square root of the dislocation density stored in the boundaries when the misoriention angle is below a certain critical value [21,23]. Therefore, the Vickers hardness (HV) can be described by the following relationship:

(1) $$HV=H_{0}+C\left[M\alpha G\sqrt{3b(1-f)\theta_{LAB}}+k\sqrt{f}\right]d^{-1/2}$$

where *H*_0_ and *C* are constants related with the hardness measurements, *M* is the Taylor factor, *α* is a numerical factor, *G* is the shear modules, *b* is the Burgers vector, *f* is the fraction of HAGBs, *θ_LAB_* is the mean misorientation angle of LAGBs, k is a constant and d is the grain size. The variation of hardness by the grain size using experimental data and Equation (1) is shown in Figure 5c. It can be clearly seen that the Vickers hardness *HV* correlates linearly with *d*^−1/2^ if all the other parameters in Equation (1) are assumed to be constants. The hardness depends on not only the average grain size but also on the microstructural features, such as the dislocation density, the mean misorientation angle, low angle boundaries and the fraction of HAGBs.

### 3.3. Microstructure Evolution during SMAT

**Figure 6:**
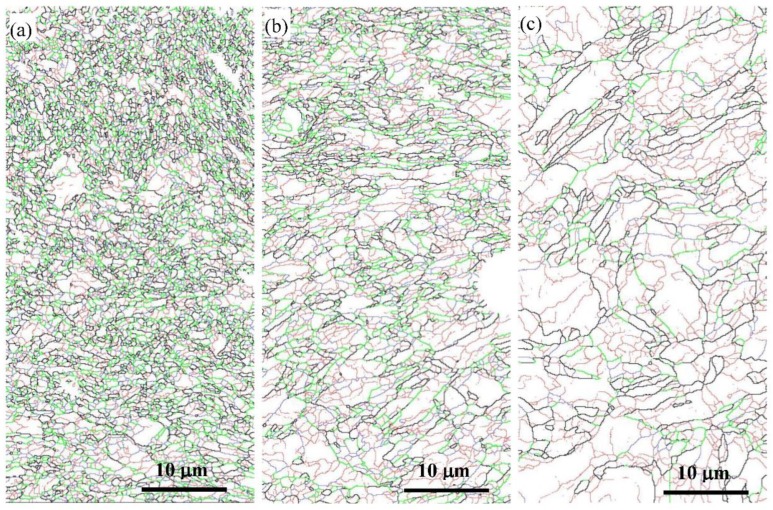
Boundary maps of the reduced activation steel after SMAT (corresponding to Figure 5): (**a**) The ultrafine grain zone, (**b**) the transition zone and (**c**) the deformed zone.

The typical EBSD orientation color maps of the reduced activation steel after SMAT are shown in Figure 5, and the boundary maps corresponding to the same regions in Figure 5 are shown in Figure 6. The different colors in Figure 5 correspond to the different crystallographic orientations normal to the observed plane [18]. The different colors in Figure 6 correspond to different misorientation, and all the lines in Figure 6b have the same meaning as those in Figure 2b.

It was reported that three different zones were observed from the EBSD maps of the graded microstructure in the surface of a 316 L stainless steel formed by SMAT [14], and there were also three zones observed from the EBSD maps in the present work. Equiaxed grains with extremely small grain sizes were observed in the surface layer (namely the UFG zone) after SMAT (Figure 5a), and most of the grain boundaries are HAGBs (Figure 6a). However, the number fraction of the boundaries with misorientation between 30° and 45° (the thick green lines) in the UFG zone were significantly larger than those before SMAT (Figure 2b). From the misorientation map, somewhat elongated regions, divided by LAGBs or dislocation boundaries, were observed in the deformed zone (Figure 6c). The initial microstructure, consisting of typical lath martensite before SMAT, were drastically destroyed in this region. Compared with the initial microstructure (Figure 2), a higher fraction of MHAGBs are seen in the deformed zone. However, nanoscale lamellae grains were observed in the transition zone (Figure 5b), and the lamellae were parallel to the treated surface of the sample. It can be seen that, with the increase of depth, the thickness of the lamellae also increased. There were more boundaries with misorientation between 30° and 45° (the thick green lines) in the transition zone than those in the deformed zone, but less than those in the UFG zones.

**Figure 7:**
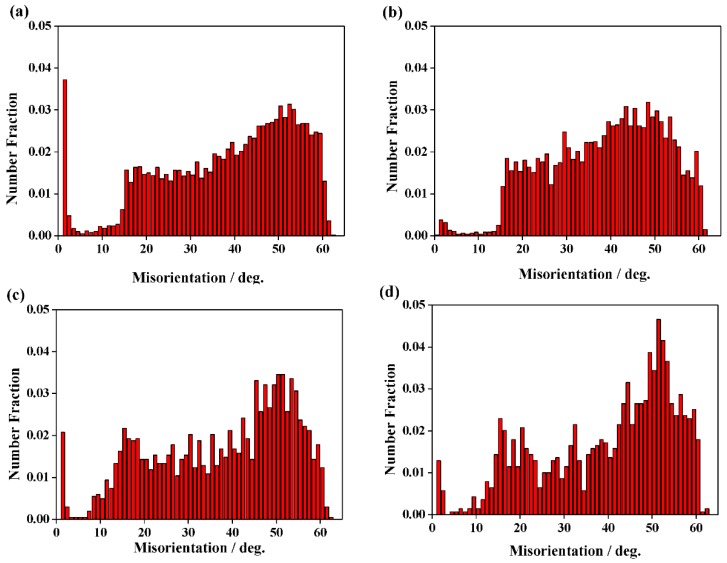
Histograms of the misorientation angles of (**a**) the ultrafine grains zone, (**b**) the transition zone, (**c**) the deformed zone after SMAT and (**d**) the initial microstructure before SMAT.

Histograms of the misorientation angles of the initial microstructure and different zones after SMAT are shown in Figure 7. Compared with the misorientation distribution of the initial microstructure (Figure 7d), a remarkable misorientation decrease can be seen in the deformed zone (Figure 7c). A remarkable increase of the number fraction of MHAGBs/MAGBs can be seen when decreasing the depth from the transition zone (Figure 7b) to the UFG zone (Figure 7a). However, the number fraction of the boundaries with orientations between 5° and 15° was extremely small in all the zones after SMAT, especially in the transition zone (Figure 7b).

In the present work, there were three different zones in the SMAT-affected layers. The UFG zone was observed at the extreme top surface, where extremely fine equiaxed grains, mostly with HAGBs, were found. While the deformation zone, where initial grains were simply deformed, was observed in the deeper region. The transition zone, where the elongated grains parallel the treated surface, was observed between the UFG zone and deformation zone. The three zones in the current work were similar to the graded microstructure in the surface of 316 L stainless steel formed by SMAT [14], and the microstructure of pure copper subjected to friction sliding deformation at room temperature [15]. However, the mechanism of the grain refinement in the present work was completely different from those used for 316 L stainless steel [14] or pure copper [15].

It is reported that there were two stages during the SMAT process, including the elastic–plastic deformation stage and the dynamic restoring stage [24]. With the increase of depth, the strain rate and strain decreased gradually, and this phenomenon correlated to the graded microstructure changes from the treated surface to the strain-free matrix. A higher strain rate and strain were more effective in producing nanograins [25]. However, due to the balance between the elastic–plastic deformation stage and the dynamic restoring stage, the depth of the nanolayer did not increase any more when the limitation of the strain and/or strain rate was reached.

Experimental results of grain refinement and microstructure evolution in pure copper subjected to SMAT showed that the mechanisms for grain refinement under SPD were related to the levels of strain rate [26]. In the region with a high strain rate (the topmost surface layer of the SMAT Cu samples), twin-matrix lamellas were firstly formed from the original coarse grains, then the twin-matrix lamellas subsequently subdivided into equiaxed nanosized blocks, and the randomly oriented nanosized grains were formed finally from the nanosized blocks. However, in the region with a low strain rate (the subsurface layer of the SMAT Cu samples), the formation of dislocation cells was regarded as the main mechanisms of the grain refinement, which can be described by the formation of sub-boundaries with small misorientations transformed from the dislocation cell walls, and the transformation of sub-GBs into highly misoriented GBs. In contrast, shear twins and bands have not been observed in other materials, such as aluminum alloy [27], iron [16] and steels [28]. 

In the present work, the mechanisms of grain refinement of ferritic/martensitic steel during SMAT were similar to that of pure Fe [16]. The dislocation activities were motivated in the original coarse grains during plastic deformation, and the dense dislocation walls (DDWs) and dislocation tangles (DTs) were formed firstly during this process. The intersecting DDWs and DTs subdivided the original ferrite grains into finer blocks (or dislocation cells) [25]. With the increase of strain, more and more dislocations formed and accumulated. After a certain strain level, dislocation rearrangement and annihilation occurred in dislocation walls (DWs). With the further increase of strains, more dislocations were generated and annihilated in the sub-grain boundaries or DWs. There was a competition between dislocation multiplication and annihilation during the whole treatment. However, the stabilized grain size was obtained when the balance was reached between the dislocation multiplication and annihilation. Finally, equiaxed nanosized grains were formed in the topmost surface layer of the sample during SMAT. However, the number fraction of MHAGBs/MAGBs after SMAT was larger than that before SMAT.

## 4. Conclusions

In summary, SMAT was used to produce a gradient NS layer at the surface of a RAFM steel, and a coupled EBSD/TEM analysis was used to investigate the microstructure evolution during SMAT. The main results are summarized as follows.

1. Both the TEM images and EBSD maps showed that there were three different zones after SMAT. The UFG zone was observed at the topmost surface, while original grains were fragmented in the “transition zone” due to the severe plastic deformation, and initial grains were simply deformed in the “deformed zone”.
2. The average grain sizes rapidly increased with the increase of depth after SMAT. The Vickers hardness in the topmost surface of the sample subjected to SMAT was the highest and decreased with the increase of depth. This phenomenon can be explained in terms of boundary strengthening and dislocation strengthening.
3. A remarkable change of misorientation distribution during SMAT was observed. When comparing the misorientation distribution after and before SMAT, a clear decrease of misorientation from the VHAGBs to MHAGBs/MAGBs was observed. However, the number fraction of the boundaries with orientations between 5° and 15° was extremely small in all the zones after SMAT, especially in the transition zone.
